# Does Electroencephalography Contribute to Examining Children with Attention Deficit Hyperactivity Disorder?

**Published:** 2014

**Authors:** Afshin FAYYAZI, Ali KHAJEH

**Affiliations:** 1 Department of Pediatric Neurolog, Hamedan University of Medical Sciences, Hamadan, Iran; 2 Department of Pediatric Neurolog, Zahedan University of Medical Sciences, Zahedan, Iran

**Dear Editor**

Attention Deficit Hyperactivity Disorder (ADHD) is the most common behavioral disorder in children (1). Prevailing theories suggest that this disease is caused by the disruption of dopamine transmission in the frontal lobe and frontostriatal pathway (2). The prevalence of such a disorder worldwide is approximately 5.2–12% (1)(2). 

ADHD is one of the most common disorders followed by epilepsy that affects the quality of the patients’ life and is an important risk factor of academic achievement (3). Many recent studies imply that there is a complex and bidirectional relationship between ADHD and epilepsy (3)(4)(5).

Previous studies have indicated a strong relationship between epilepsy and neuropsychological disorders such as Electrical Status Epilepticus in Sleep (ESES) and Landau–Kleffner syndrome (LKS) (1) (4). Epilepsy in children is associated with behavioral problems, such as ADHD and cognitive impairments. Cognitive disabilities are more likely to worsen with early onset and uncontrolled epilepsy. 

Children with some of the epileptic syndromes such as benign childhood epilepsy with centrotemporal spikes (BCECTS), frontal lobe epilepsy, rolandic epilepsy, complex partial seizures (CPS), and absence seizures may be more likely to be associated with these symptoms(6). 

Epidemiological studies have found that children with epilepsy are more susceptible to ADHD than other children are, so that one-third to two-third of such children will be affected by this disorder (3)(5). Incidences of ADHD in epileptic children will increase due to existing factors such as gender (male), positive family history, mental retardation, frontal lobe damage, intractable seizures, subclinical epileptic waves in interictal phase, focal seizures of frontal and temporal lobe as well as the use of anticonvulsant drugs including Phenobarbital, Benzodiazepine, Topiramate, and Vigabatrin (5).

Other studies have shown that ADHD has a higher risk of incidence in generalized seizures than spasms. In some epileptic syndromes, ADHD is observed as a high risk, so that 67% of the patients with frontal lobe epilepsy and 61% with childhood absence epilepsy had ADHD (3). ESES is strongly associated with the behavioral disorders such as ADHD (3).

On the other hand, the results of other studies have indicated the presence of epileptic discharges in some children suffering from ADHD without a history of epilepsy. In consideration of this evidence, a causal relationship between subclinical epileptic waves and occurrence of symptoms is raised (2) (4) (5) (7) (8). In other studies, it has been found that if such abnormal waves are treated, symptoms associated with ADHD will recover (4)(9). Mason et al conducted a study of 10 patients with cognitive impairments showing epileptic waves in EEGs and were treated by anticonvulsant drugs. The study indicated that 80% had recovered (9). On the other hand, Carbamazepine is used as a non-FDA approved off-label medication for ADHD in non-epileptic children as an alternative to stimulants (6).

Fonseca et al and related research found that Epileptiform Discharges were found in 10% of ADHD patients. The ADHD group showed significantly greater absolute delta and theta powers and also greater absolute beta power and smaller relative alpha 1 and beta powers at some electrodes when compared to the controls (10). 

The increased slow-wave and reduced fast-wave activity commonly reported in the ADHD literature (11).Richer et al conducted a study with EEGs performed on 347 patients with ADHD to reveal that 6.1±1.3% of patients had epileptic form waves, which was significantly different from the control group (3.5±0.6%) where p <0.025. In this investigation, all cases with seizures and other brain disorders were excluded (8).Holtmann et al conducted EEGs on 483 patients suffering from ADHD without a history of seizure, of which 5.6% of the patients showed rolandic waves, which is significantly different from the control group with 2.4% where p <0.001 (7).Hughes et al carried out a study of that examined 176 patients through EEGs that showed 30.1% of cases had epileptic waves, 6% of which were two-way waveforms-acupuncture waves (12). A recent study found one in four non-epileptic children evaluated for ADHD has epileptiform discharges in EEGs during sleep and sleep deprivation, in comparison to 7% in wake only records(13).

This phenomenon can be justified by two hypotheses: 1. epilepsy and behavioral disorders may be associated with an underlying genetic disorder and considered a variety of manifestations of a neurologic disorder (7); and, 2. the incidence of behavioral disorder as related to behavioral manifestations of subclinical epileptic waves that can be improved by use of anticonvulsant drugs (7) & (9). Some investigations have used the term Transient Cognitive Impairment (TCI) for secondary cognitive disorders affected by epileptic waves (12).

Unlike the studies emphasizing the performance of EEG in the patients with ADHD, other investigations have suggested the excessive usage of EEG in these patients (11). Matoth et al concluded that, after seizure, ADHD is the second reason for using EEG in 561 children referred to as ADHD, among which 94% had normal, 1% had slow, and 5% had epileptic waves. In this study, the results obtained from EEG on ADHD patients were not different from normal children. Thus, EEG is not suggested by this study as an appropriate method to examine the children with ADHD (14). 

In patients with ADHD and seizures controlled by antiepileptic medication, stimulant therapy is generally safe. When stimulant therapy is introduced for patients with epilepsy or subclinical electrographic abnormalities not treated with anticonvulsants are at increased risk of seizures (13).

Some of studies have demonstrated that strengthening sensorimotor rhythm waves while inhibiting theta waves by EEG feedback is effective in the treatment of ADHD(15) (16).

Considering the literature related to this issue, it seems essential to have a logical approach to the use of EEG. 

On one hand, EEG may contribute to diagnosis of the disease in the patients suffering from ADHD, especially when Deficit Attention is dominant (3). On the other hand, given that the result of EEG is normal in most of the cases, it seems logical to prevent excessive use. 

Therefore, it is recommended to perform EEG on patients suffering from ADHD with a high risk in epileptic areas, but not for all patients. According to some resources, the high-risk groups have the following features and symptoms: a history of previous seizures, prenatal events, a history of head trauma, variable behavioral manifestations, and other existing cognitive disorders such as mental retardation as well as a family history of seizures (1) (7). The use of anticonvulsant drugs in case of clear epileptic waves in children who suffer from ADHD is recommended for controlling cognitive symptoms (7) (9).
